# *Mesoplasma florum*: a near-minimal model organism for systems and synthetic biology

**DOI:** 10.3389/fgene.2024.1346707

**Published:** 2024-02-09

**Authors:** Dominick Matteau, Anthony Duval, Vincent Baby, Sébastien Rodrigue

**Affiliations:** ^1^ Département de biologie, Université de Sherbrooke, Sherbrooke, QC, Canada; ^2^ Centre de diagnostic vétérinaire de l’Université de Montréal, Université de Montréal, Saint-Hyacinthe, QC, Canada

**Keywords:** *Mesoplasma florum*, Mollicutes, synthetic biology, systems biology, minimal genome

## Abstract

*Mesoplasma florum* is an emerging model organism for systems and synthetic biology due to its small genome (∼800 kb) and fast growth rate. While *M. florum* was isolated and first described almost 40 years ago, many important aspects of its biology have long remained uncharacterized due to technological limitations, the absence of dedicated molecular tools, and since this bacterial species has not been associated with any disease. However, the publication of the first *M. florum* genome in 2004 paved the way for a new era of research fueled by the rise of systems and synthetic biology. Some of the most important studies included the characterization and heterologous use of *M. florum* regulatory elements, the development of the first replicable plasmids, comparative genomics and transposon mutagenesis, whole-genome cloning in yeast, genome transplantation, in-depth characterization of the *M. florum* cell, as well as the development of a high-quality genome-scale metabolic model. The acquired data, knowledge, and tools will greatly facilitate future genome engineering efforts in *M. florum*, which could next be exploited to rationally design and create synthetic cells to advance fundamental knowledge or for specific applications.

## Introduction

Mollicutes form a group of bacteria characterized by the absence of a cell wall and exceptionally small genomes. During the past decades, the field of molecular and cellular biology experienced significant advances, leading to a heightened interest for this class of bacteria. As new molecular data was generated, more particularly about the mycoplasmas, the idea that these microorganisms could actually be the simplest self-replicating life forms existing on Earth was becoming increasingly plausible (Morowitz and Tourtellotte, 1962; Morowitz, 1984). The minimal genome concept started to emerge: what is the smallest set of genes required for autonomous life, and what functions do they encode? Are there many or only one possible combination of genes composing a minimal genome? If we could understand the function of every single gene in a cell, we would have a better comprehension of the most fundamental principles of life (Peterson et al., 2001; Glass et al., 2017; Lachance et al., 2019). Just as the study of the hydrogen atom was fundamental in developing the laws of quantum physics, examining the simplest autonomous cells presented itself as the most logical starting point for this endeavor (Morowitz and Tourtellotte, 1962; Morowitz, 1984). An impressive number of Mollicutes species were isolated during the 1980s and 1990s, including many species associated with plants and insects (Whitcomb and Tully, 1995; Pettersson and Johansson, 2002). Unlike most mycoplasmas, which are typically parasitic, many of these species appeared to be commensals, coexisting in a mutually beneficial relationship with a variety of animal hosts. Many of these isolates also showed no strict requirement of sterols or cholesterol for growth *in vitro*, and were initially regrouped under the genus name *Acholeplasma* (Tully, 1979; Tully, 1983; Tully et al., 1990; Tully et al., 1993). This was the case for *Mesoplasma florum*, a bacterium that has become an interesting model organism for the fields of systems and synthetic biology.

## What is *Mesoplasma florum*?

**FIGURE 1:**
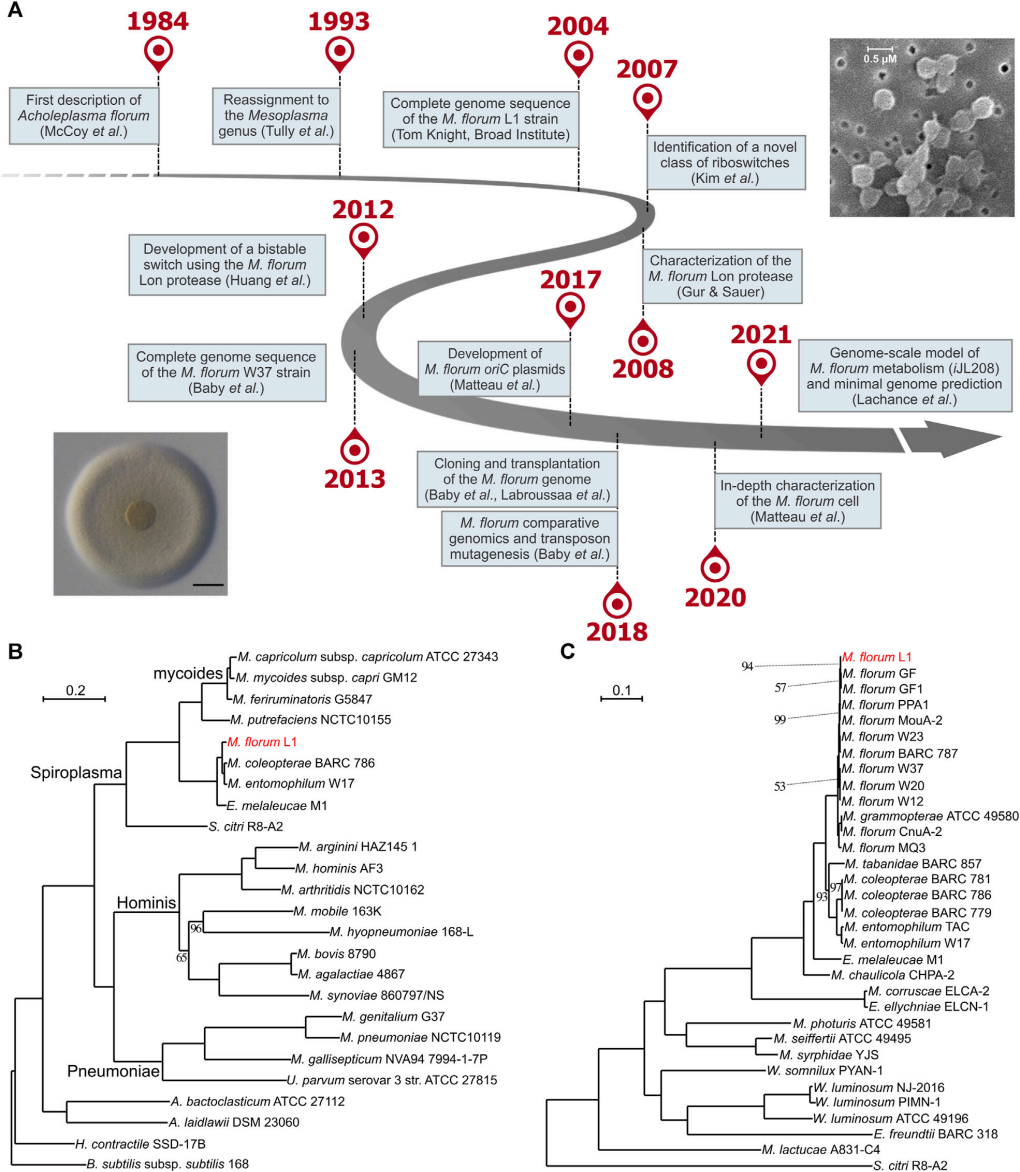
Forty years of research on *Mesoplasma florum*. **(A)** Important milestones in *M. florum* research timeline. Representative picture of an *M. florum* L1 colony displaying the typical “fried-egg” morphology (adapted from Labroussaa et al., 2016, Vol. 44, No. 17, pp. 8501–8511, by permission of Oxford University Press; scale bar: 100 µm) as well as *M. florum* L1 cells observed by scanning electron microscopy (Baby et al., 2013) are also depicted. **(B)** and **(C)** Maximum-likelihood phylogenetic trees of the Mollicutes **(B)** and the *Mesoplasma/Entomoplasma* genera **(C)** inferred using concatenated alignments of 109 and 229 conserved proteins, respectively. Trees were constructed using RAxML (Stamatakis, 2014) with 150 bootstrap replicates as determined using the autoFC bootstopping criterion. Bootstrap replicate values are of 100 unless specified otherwise. *Bacillus subtilis* and *S. citri* were used as outgroups. See Table 1 for additional information on strains and genomes included in the *Mesoplasma/Entomoplasma* phylogenetic tree.

*M. florum* is a small (0.5–0.6 µm), ovoid, near-minimal and non-pathogenic bacterium of the Mollicutes class (Figure 1A) initially described for the first time as *Acholeplasma florum* in 1984 by McCoy and colleagues (McCoy et al., 1984). The species was named after its recovery site‐the flowers of healthy plants found in Florida, United States. *M. florum* L1, the type strain of the species, was isolated from flowers of a lemon tree (*Citrus limon*) (McCoy et al., 1980; McCoy et al., 1984). Since *M. florum* grew in culture media without sterols it was originally classified in the genus *Acholeplasma* (Tully, 1979; Tully, 1983; Clark et al., 1986; Tully et al., 1990). However, this species was reassigned to the *Mesoplasma* genus in 1993 according to new physiological and molecular evidence, including phylogenetic clustering based on 16S rRNA sequence analysis (Tully et al., 1993). *M. florum* is in fact closely related to a phylogenetically distinct group of mycoplasmas called the mycoides cluster (Figure 1B). This cluster notably includes *Mycoplasma mycoides* and *Mycoplasma capricolum*, two well-known model organisms for the fields of systems and synthetic biology (Sirand-Pugnet et al., 2007; Glass et al., 2017; Lachance et al., 2019). Yet, in contrast to *M. mycoides* and *M. capricolum*, *M. florum* has never been associated with any disease, and no virulence factor has been identified in its genome. As for other members of the class Mollicutes, *M. florum* does not have a cell wall and its genome is particularly small, varying from 738,512 (BARC 787) to 830,640 bp (W20) depending on the exact strain, with an average GC content of about 27% (Baby et al., 2018b). *M. florum* genes are predominantly oriented according to the direction of DNA replication, frequently expressed as polygenic transcriptional units, and occupy most of the genome space, typical of bacterial genomes (Baby et al., 2018b; Matteau et al., 2020). This bacterium also uses an alternative genetic code (the *Mycoplasma* and *Spiroplasma* code) in which the canonical UGA stop codon rather codes for the incorporation of a tryptophan (Navas-Castillo et al., 1992). This distinctive feature is also present in mycoplasmas of the mycoides cluster as well as in the phylogenetically related Mollicute *Spiroplasma citri*, the causative agent of the Citrus stubborn disease (Saglio et al., 1973).

Among all previously isolated *M. florum* strains, the L1 strain is the most extensively studied. Compared to most Mollicutes, *M. florum* L1 shows a remarkably fast growth rate, corresponding to a doubling time of ∼32 min at the optimal growth temperature (34°C) (Matteau et al., 2020). In comparison, *M. mycoides* subspecies *capri* has a doubling time of ∼60 min in similar conditions (Gibson et al., 2010; Hutchison et al., 2016), whereas for *M. capricolum* subspecies *capricolum* and *Mycoplasma pneumoniae* this value is estimated to be around 90 min and 8–20 h, respectively (Seto and Miyata, 1998; Yus et al., 2009; Wodke et al., 2013). Since Mollicutes have experienced massive gene loss events through evolution, they have lost the capacity to synthesize many metabolites, resulting in an important simplification of their metabolism (Sirand-Pugnet et al., 2007). In *M. florum,* for example, most of the biosynthesis occurs through salvage pathways, and the energy production relies exclusively on glycolysis and fermentation since no respiratory system is present (Lachance et al., 2021). Consequently, this bacterium, as for most Mollicutes, requires a very rich medium to palliate its metabolic deficiencies *in vitro*. The most common growth medium for *M. florum* is the ATCC 1161, a complex and undefined medium containing horse serum, yeast extract, and heart infusion broth. Other similar media such as SP5 have also been used (Whitcomb et al., 1982; McCoy et al., 1984; Pollack and Williams, 1996; Matteau et al., 2015; Matteau et al., 2020; Baby et al., 2018a). *M. florum* L1 colonies display the typical Mollicutes “fried-egg” appearance on solid medium (Figure 1A) (McCoy et al., 1984; Tully et al., 1994; Labroussaa et al., 2016), and batch cultures growing in ATCC 1161 display the four typical bacterial growth phases (lag, exponential, stationary, and decline), reaching up to ∼10^10^ cells/mL at the end of the exponential growth phase (Matteau et al., 2015; Matteau et al., 2020). *M. florum* growth rate is however highly limited by the concentration of horse serum and yeast extract present in the medium, clearly demonstrating the dependance of this bacterium on pre-assembled building blocks for its metabolism (Lachance et al., 2021). The end of the exponential phase also coincides with an important drop in the pH of the medium, most likely due to the accumulation of lactate and acetate fermentation products (Pollack and Williams, 1996; Matteau et al., 2020; Lachance et al., 2021). This decrease in the medium’s pH is likely to be responsible for the progressive death of the *M. florum* cell population after the stationary phase. Indeed, no significant mortality is observed when the exponential phase is maintained using a continuous culture device (Matteau et al., 2015).

## Where does *M. florum* primarily live?

*M. florum* is hypothesized to live primarily inside the gastrointestinal tract of insects, which would provide continuous access to complex nutrients such as sugars, lipids, peptides, and other metabolites required for growth. The continuous flow of the digestive tract would also prevent the accumulation of fermentation products and the possible acidification of the milieu, acting similar to a continuous culture device (Matteau et al., 2015). This natural habitat would also explain the presence of this bacterium on plant surfaces as insects would carry them from site to site and excrete the microbe through their feces (Whitcomb et al., 1982; McCoy et al., 1984; Tully et al., 1990). The extracellular polysaccharide layer surrounding *M. florum* cells, which was shown to occupy for up to 5% of the total *M. florum* biomass, probably contributes to the survivability of this microorganism on plant surfaces (Matteau et al., 2020; Lachance et al., 2021). Mainly composed of galactose and glucose, this capsule-like structure might provide a physical protection against desiccation, and therefore participate in the dissemination of *M. florum* across insect populations. The possibility that *M. florum* uses plants as secondary hosts like some pathogenic spiroplasmas seems rather unlikely since no such observation has ever been reported and *M. florum* has never been isolated in the context of a plant disease.

The full range of hosts susceptible to *M. florum* colonization and the possibility of a predominant association with specific insect types are still not well-defined. Although a few strains were directly recovered from the gut content of insects such as soldier beetles (*Cantharidae*) as well as Vespid wasps (*Monobia quadridens*) (Clark et al., 1986; Tully et al., 1987), most of the previously described *M. florum* strains were originally isolated from plant flowers (Table 1). This prevents their direct association with an insect host. Still, the isolation source of closely related species, especially species of the *Mesoplasma* and *Entomoplasma* genera, suggests that *M. florum* could potentially be found in a wide variety of insects, including firefly beetles (*Ellychnia corrusca*), goldenrod soldier beetles (*Chauliognathus pennsylvanicus*), as well as tabanid (*Tabanus catenatus*) and syrphid (*Syrphidae*) flies (Clark et al., 1986; Tully et al., 1987; Tully et al., 1989; Tully et al., 1994). Furthermore, *Mesoplasma* and *Entomoplasma* have intermixed relationships and recent phylogenetic data suggest that they should no longer be taxonomically separated (Gasparich and Chih-Horng, 2019) (Figure 1C).

**TABLE 1:** List of *Mesoplasma* and *Entomoplasma* strains with genome assemblies deposited on the RefSeq database.

Organism name	Previous names	Strain name	Original reference	Isolation source	Source details	RefSeq accessiona	Submitter	Submission date	Assembly level	Length (bp)
*Entomoplasma ellychniae*	*Mycoplasma ellychniae*	ELCN-1	Tully et al. (1989)	*Ellychnia corrusca*	Hemolyph of firefly beetle	GCF_002930155.1	Academia Sinica	15/02/2018	Contig	900,037
*Entomoplasma freundtii*	-	BARC 318 (ATCC 51999)	Tully et al. (1998)	*Coleoptera Cicindelidae*	Green tiger beetle gut tissue	GCF_002804205.1	Academia Sinica	04/12/2017	Complete	838,114
*Entomoplasma melaleucae*	*Mycoplasma melaleucae*	M1 (ATCC 49191)	Tully et al. (1990b)	*Melaleuca quinquenervia*	Surface of tropical plant flower	GCF_002804105.1	Academia Sinica	04/12/2017	Complete	845,295
*Mesoplasma chauliocola*	-	CHPA-2 (ATCC 49578)	Tully et al. (1987); Tully et al. (1994)	*Chauliognathus pennsylvanicus*	Gut of goldenrod solider beetle	GCF_002290085.1	Ginkgo Bioworks Inc	12/09/2017	Complete	854,780
*Mesoplasma coleopterae*	-	BARC 779 (ATCC 49583)	Tully et al. (1994)	*Chauliognathus sp*	Gut of adult soldier beetles	GCF_002804245.1	Academia Sinica	04/12/2017	Complete	800,407
*Mesoplasma coleopterae*	*Mesoplasma florum*	BARC 781	Unpublished	Beetle	-	GCF_002999455.1	Universite de Sherbrooke	14/03/2018	Chromosome	803,948
*Mesoplasma coleopterae*	*Mesoplasma florum*	BARC 786	Unpublished	Beetle	-	GCF_002999395.1	Universite de Sherbrooke	14/03/2018	Chromosome	765,660
*Mesoplasma corruscae*	-	ELCA-2 (ATCC 49579)	Tully et al. (1987); Tully et al. (1994)	*Ellychnia corrusca*	Gut of an adult firefly beetle	GCF_002930145.1	Academia Sinica	15/02/2018	Contig	839,085
*Mesoplasma entomophilum*	*Acholeplasma entomophilum*	TAC (ATCC 43706)	Clark et al. (1986)	*Tabanus catenatus*	Gut of tabanid fly	GCF_002749675.1	Ginkgo Bioworks Inc	03/11/2017	Complete	847,967
*Mesoplasma entomophilum*	*Mesoplasma florum*; *Acholeplasma florum*; *Acholeplasma entomophilum*	W17	Whitcomb et al. (1982)	*Aster sp*	Surface of plant flower	GCF_002999315.1	Universite de Sherbrooke	14/03/2018	Chromosome	787,107
*Mesoplasma florum*	*Acholeplasma florum*	L1 (ATCC 33453)	McCoy et al. (1984)	*Citrus limon*	Surface of plant flower	GCF_000008305.1	Broad Institute	19/07/2004	Complete	793,224
*Mesoplasma florum*	*Acholeplasma florum*; *Acholeplasma entomophilum*	W37	Whitcomb et al. (1982)	*Solidago sp*	Surface of plant flower	GCF_000479355.1	Universite de Sherbrooke	24/10/2013	Complete	825,824
*Mesoplasma florum*	*Acholeplasma florum*	GF1	Whitcomb et al. (1982)	*Citrus limon*	Surface of plant flower	GCF_002504365.1	Ginkgo Bioworks Inc	10/10/2017	Complete	807,195
*Mesoplasma florum*	-	PPA1	Unpublished	*Calliandra haematocephalus*	Surface of plant flower	GCF_002504385.1	Ginkgo Bioworks Inc	10/10/2017	Complete	820,043
*Mesoplasma florum*	-	BARC 787	Unpublished	Unspecified insect	-	GCF_002999435.1	Universite de Sherbrooke	14/03/2018	Complete	738,512
*Mesoplasma florum*	*Acholeplasma florum*	CNUA-2	Tully et al. (1987)	*Coleoptera: Cantharidae*	Gut of soldier beetle	GCF_002999275.1	Universite de Sherbrooke	14/03/2018	Complete	813,801
*Mesoplasma florum*	-	GF	Unpublished	-	-	GCF_002999355.1	Universite de Sherbrooke	14/03/2018	Chromosome	792,347
*Mesoplasma florum*	-	MouA-2	Unpublished	*Monobia quadridens*	Vespid wasp	GCF_002999255.1	Universite de Sherbrooke	14/03/2018	Complete	781,099
*Mesoplasma florum*	*Acholeplasma florum*	MQ3 (MQ-3)	Clark et al. (1986)	*Monobia quadridens*	Gut of a Vespid wasp	GCF_002999415.1	Universite de Sherbrooke	14/03/2018	Complete	793,277
*Mesoplasma florum*	*Acholeplasma florum*	W20	Whitcomb et al. (1982)	*Aster simplex*	Surface of plant flower	GCF_002999375.1	Universite de Sherbrooke	14/03/2018	Chromosome	830,640
*Mesoplasma florum*	*Acholeplasma florum*	W23	Whitcomb et al. (1982)	*Helianthus annuus*	Surface of plant flower	GCF_002999295.1	Universite de Sherbrooke	14/03/2018	Complete	773,885
*Mesoplasma florum*	*Acholeplasma florum*	W12	Whitcomb et al. (1982)	*Chrysothamnus sp*	Surface of plant flower	GCF_003006095.1	Universite de Sherbrooke	16/03/2018	Chromosome	829,202
Mesoplasma grammopterae	-	GRUA-1 (ATCC 49580)	Tully et al. (1987); Tully et al. (1994)	*Grammoptera sp*	Gut of adult long-horned beetle	GCF_000701525.1	DOE Joint Genome Institute	11/06/2014	Scaffold	806,944
Mesoplasma lactucae	*Mycoplasma lactucae*	831-C4 (ATCC 49193)	Rose et al. (1990)	*Lactuca sativa*	Surface of lettuce plant	GCF_002441935.1	Ginkgo Bioworks Inc	04/10/2017	Complete	837,471
*Mesoplasma photuris*	-	PUPA-2 (ATCC 49581)	Tully et al. (1987); Tully et al. (1994)	*Photuris sp*	Gut of firefly larva	GCF_000702725.1	DOE Joint Genome Institute	11/06/2014	Contig	778,966
*Mesoplasma seiffertii*	*Acholeplasma seiffertii*	F7 (ATCC 49495)	Bonnet et al. (1991)	*Citrus senensis*	Surface of sweet orange flower	GCF_000518725.1	DOE Joint Genome Institute	13/01/2014	Scaffold	977,957
*Mesoplasma syrphidae*	-	YJS (ATCC 51578)	Tully et al. (1994)	*Diptera: Syrphidae*	Gut of an adult yellowjacket-like syrphid fly	GCF_002843565.1	Ginkgo Bioworks Inc	17/12/2017	Complete	908,214
*Mesoplasma tabanidae*	-	BARC 857 (ATCC 49584)	Tully et al. (1994)	*Tabanus abactor*	Gut of adult horse fly	GCF_002804025.1	Academia Sinica	04/12/2017	Complete	846,907
*Williamsoniiplasma lucivorax*	*Entomoplasma lucivorax*; *Mycoplasma lucivorax*	PIPN-2 (ATCC 49196)	Williamson et al. (1990)	*Photinus pyralis*	Gut of an adult firefly beetle	GCF_000518285.1	DOE Joint Genome Institute	13/01/2014	Scaffold	11,03,092
*Williamsoniiplasma luminosum*	*Entomoplasma luminosum*; *Mycoplasma luminosum*	PIMN-1 (ATCC 49195)	Williamson et al. (1990)	*Photinus marginalis*	Gut of an adult firefly beetle	GCF_002803985.1	Academia Sinica	04/12/2017	Complete	10,31,560
*Williamsoniiplasma luminosum*	*Entomoplasma luminosum*; *Mycoplasma luminosum*	NJ-2016	Unpublished	*Photinus pyralis*	Gut of an adult firefly beetle	GCA_003013295.1	Photinus pyralis genome working group	21/03/2018	Complete	10,29,845
*Williamsoniiplasma somnilux*	*Entomoplasma somnilux*; *Mycoplasma somnilux*	PYAN-1 (ATCC 49194)	Williamson et al. (1990)	*Pyractonema angulata*	Pupal gut of the firefly beetle	GCF_002804005.1	Academia Sinica	04/12/2017	Complete	868,413

aWhen more than one genome assemblies were available, the most complete assembly was selected. If assembly levels were identical, then the first submitted assembly was chosen.

While we cannot completely rule out the possibility that *M. florum* could be pathogenic in certain hosts or under yet unidentified circumstances, its ecological niche seems quite different from related pathogenic mycoplasmas of the mycoides cluster. Since the growth of *M. florum* is dramatically impaired at 37°C (McCoy et al., 1984; Matteau et al., 2020), the probability that it infects warm-blooded animals similar to *M. mycoides* or *M. capricolum* is indeed very low. Recent data suggest that mycoplasmas of the mycoides cluster rather gained the ability to infect animals like other mycoplasmas through convergent evolution, in which a common ancestor experienced important gene losses and acquisitions, notably by exchanging genes with the Hominis and Pneumoniae lineages (Lo et al., 2018). Whether *M. florum* simply benefits from its hosts or rather perform advantageous metabolic activities, for example, by degrading or secreting particular metabolites in the gut, remains also to be determined. It has been shown that some bacteria of the *Entomoplasmatales* clade play important roles in the digestive system of attine fungus-farming leaf-cutting ants (Sapountzis et al., 2015; Sapountzis et al., 2018). In any cases, *M. florum* or its predecessor had to adapt and develop strategies to compete for the available resources. Its small size might in fact be advantageous in that context. With an average cell diameter of 0.5–0.6 µm (Figure 1A), *M. florum* is estimated to have a total cell volume of only 0.08–0.10 µm^3^, which is nearly 50 times smaller than *Escherichia coli* (Volkmer and Heinemann, 2011; Dai and Zhu, 2018; Matteau et al., 2020). This causes *M. florum* cells to have a surface area to volume ratio approximately 2.5 times higher than *E. coli*, as well as a relatively higher biomass fraction allocated to lipids (∼18%). These characteristics probably facilitate the importation of complex nutrients from the environment that are required for biosynthesis reactions and ATP production. Given its scavenger lifestyle, nutrient acquisition certainly occupies a critical role in *M. florum* metabolism. Transport reactions actually represent about a third (84/277) of the total number of reactions included in the recently published genome-scale model (GEM) of *M. florum* (Lachance et al., 2021). This is also reflected by the capacity of *M. florum* L1 to import and process various sugars, including glucose, fructose, sucrose, trehalose, and maltose (Lachance et al., 2021). Since the glycolysis is the only way of producing ATP in *M. florum*, being able to degrade various sugars might be important to survive in the insect gut, especially if the hosts diet is variable across individuals or between feeding periods. Interestingly, genes responsible for carbohydrate transport and metabolism are among the most variable between *M. florum* strains (Baby et al., 2018b), suggesting that some strains might be more fit to certain diets. Since the phylogeny of those strains could not be linked to their geographical origin or isolation source (Baby et al., 2018b), nutritional preferences of *M. florum* primary hosts could be one of many important actors driving the evolution of this species.

Another important consideration about very small cells is the limited amount of material that their volume can accommodate. This is well exemplified by the very small genomes of Mollicutes, which can be as small as 580 kbp in the case of *Mycoplasma genitalium* (Su and Baseman, 1990; Fraser et al., 1995). At 0.5–0.6 µm of diameter, *M. florum* cells are in fact only 5,000 to 6,000 times larger than a hydrogen atom, and weight just about 100 fg (Morowitz and Tourtellotte, 1962; Morowitz, 1984; Sundararaj et al., 2004; Matteau et al., 2020). With only ∼800 kbp, the *M. florum* chromosome obviously requires fewer nucleotides and most probably less energy than for *E. coli* to replicate, especially since both organisms have approximately the same number of genome copies per cell (Bionumbers, 2015; Matteau et al., 2020). The number of RNA and protein molecules is also much lower in *M. florum* compared to *E. coli*, corresponding to roughly 10 times fewer molecules per cell for both constituents. Yet, if we normalize these values per unit of volume, *M. florum* and *E. coli* show similar proteins and RNA concentrations (Sundararaj et al., 2004; Milo, 2013; Bionumbers, 2015; Matteau et al., 2020). Combined with the low metabolic cost predicted for *M. florum* biomass synthesis reactions, which are mainly fueled by the import, assembly, and rearrangement of premade molecular building blocks, these physical limitations might decrease the amount of energy needed to complete a round of cellular division. This probably contributes to the fast growth rate of *M. florum*, and could explain why little amounts of sugars are sufficient to sustain its growth *in vitro* (Lachance et al., 2021). The main protease responsible for the degradation of incomplete proteins that are expressed from mRNA lacking stop codons is also 8 to 16 times more processive in *M. florum* compared to *E. coli* (Gur and Sauer, 2008). This could allow a more efficient recycling of the amino acids incorporated into incomplete proteins. This protease (Lon) was notably used in metabolic engineering applications (Zhou et al., 2023) as well as to develop artificial gene circuits in other bacteria (Huang et al., 2012; Cameron and Collins, 2014; Sakkos et al., 2021; Szydlo et al., 2022). Of course, other factors most likely come into play to explain the fast-growing phenotype of *M. florum* compared to other Mollicutes. Not spending resources and energy on the expression of virulence factors is probably one of them. Allocating most of its resources on protein expression might also help, as nearly half of all protein molecules present in the *M. florum* cell are associated with translation and other related processes (Matteau et al., 2020; Lachance et al., 2021). More precisely, the estimated ribosome concentration in *M. florum* is roughly ten times higher than the values reported for *M. pneumoniae*, but comparable to concentrations estimated in *M. mycoides* and *E. coli* (Sundararaj et al., 2004; Kühner et al., 2009; Yus et al., 2009; Bakshi et al., 2012; Wodke et al., 2013; Breuer et al., 2019; Matteau et al., 2020). Rather than adopting complex survival strategies like *M. pneumoniae* and other slow-growing pathogenic mycoplasmas, *M. florum* appears to focus on rapid biomass production to thrive in its natural environment. The reconstruction of a GEM that accounts for protein expression constraints (ME-model) (Lloyd et al., 2018) and its comparison with protein abundances previously estimated for *M. florum* might provide additional clues on the relationship between protein allocation and growth rate in Mollicutes.

## Is the genome of *M. florum* minimal?

Although the *M. florum* genome has been streamlined by evolution (Sirand-Pugnet et al., 2007), previous studies showed that it is not minimal, at least not under laboratory conditions (Baby et al., 2018b; Lachance et al., 2021). Even if Mollicutes have some of the smallest genomes found in nature, a considerable fraction of their genome is dispensable in rich media. Most non-essential elements consist of genes or regulatory elements important for fitness and robustness of the cells in their natural habitat, which generally provide much more challenging and variable physicochemical conditions compared to laboratory settings. In *M. genitalium*, for example, approximately 100 of its 485 predicted protein-coding genes were found to be non-essential using random transposition mutagenesis experiments (Hutchison et al., 1999; Glass et al., 2006). Another good example is JCVI-syn3.0, the currently closest approximation of a minimal organism (Hutchison et al., 2016). This artificial bacterium harbors a synthetic chromosome of only 531 kbp and 438 protein-coding genes based on the *M. mycoides* subspecies *capri* genome, which represents an impressive reduction of roughly 50% compared to the original sequence. Still, around 25% of the remaining genes in JCVI-syn3.0 and derivative strains are of unknown function (Hutchison et al., 2016; Glass et al., 2017; Breuer et al., 2019), highlighting our current gap of knowledge in the biology of even the simplest forms of life.

What could be the *M. florum* minimal genome, and would it be any different from JCVI-syn3.0? In *M. florum*, essential genes have been studied using two different but complementary methods, i.e., comparative genomics and random transposon mutagenesis (Baby et al., 2018b). By comparing the genomic sequence of 13 *M. florum* strains, two main groups were revealed, one comprising most of the *M. florum* representatives (10/13), and a second one containing only three strains, namely, W17, BARC 781, and BARC 786. Interestingly, these three strains were recently renamed based on their average nucleotide identity with other *Mesoplasma* species (Table 1). Nonetheless, the genomes of W17, BARC 781, and BARC 786 were found to be highly syntenic with the other representatives, and a core set of 546 homologous gene cluster families was observed in all compared genomes (Baby et al., 2018b). This corresponds to approximately 80% of all protein coding genes present in each strain, which was found to vary between 651 and 740 among strains. Unsurprisingly, more than 25% of the conserved *M. florum* genes are related to translation, a functional category that was observed to be significantly enriched in the core genome compared to the entire gene sets. Still, transposon mutagenesis performed in the *M. florum* L1 strain showed that a total of 430 genes out of 720 can be interrupted by transposon, including 320 core genes (Baby et al., 2018b). No transposon was observed in the remaining 290 genes, which are most likely essential in *M. florum* L1 or could have been missed given the transposon insertion density of the study. The number of putatively essential genes was however increased to 332 upon re-analysis of the transposition insertion data by considering the insertion position of the transposons within *M. florum* open-reading frames (Lachance et al., 2021). All analyzed genomes were predicted to encode 29 tRNA genes, as well as two virtually identical copies of the rRNA gene loci, although one copy is probably sufficient for growth (Asai et al., 1999; Hutchison et al., 2016).

Gene conservation and essentiality data have been used to propose minimal genome scenarios for *M. florum* L1. One scenario would be to remove all non-core genes from its genome, which should yield a ∼645 kbp genome coding for 585 genes if all intergenic and non-coding elements are retained (Baby et al., 2018b). However, 25 non-core protein coding genes were identified to be essential for *M. florum* L1 in ATCC 1161 medium. Including these genes in the minimal genome design would thus increase the chances of producing a viable cell. The 110 genes interrupted by transposons and absent from the core genome thus represent interesting first-step candidates for genome streamlining. Yet, this genome would probably be far from minimal since a majority (∼55%) of core genes can be interrupted by transposons without severely impacting *M. florum* growth. On the other hand, keeping only the genes in which no transposon was detected is a dubious strategy since synthetic lethality interactions are likely to occur, resulting in a non-viable cell when certain combinations of genes are simultaneously deleted. Given the phylogenetic proximity between *M. florum* and *M. mycoides* (Figure 1B), another possible scenario would be to include the 409 *M. florum* L1 protein-coding genes in which an ortholog was found in JCVI-syn3.0. Intriguingly, this Syn3.0 inspired minimal genome would contain 401 of the 585 *M. florum* L1 core genes, but would lack 57 genes identified as essential in *M. florum* (Baby et al., 2018b). Conversely, 69 gene families unique to *M. mycoides* JCVI-syn3.0 would not be present in that design.

Even if we combine the 57 essential genes found only in *M. florum* L1 with the 409 protein-coding genes shared between *M. florum* and JCVI-syn3.0, it remains difficult to predict if this synthetic design will be viable. Genome design rules remain poorly understood, and most synthetic genome projects rely on trial-and-error approaches, involving long and fastidious rounds of optimization. For instance, to create JCVI-syn3.0, it took not only many rounds of genome design, transposon mutagenesis, and debugging, but also an extensive knowledge of the biochemical data available in the literature as well as an impressive amount of time and resources (Sleator, 2010; Sleator, 2016; Hutchison et al., 2016). Systems biology approaches that can integrate multiple layers of information and systematically evaluate genome designs represent promising tools in that context (Chalkley et al., 2019; Rees-Garbutt et al., 2020a; Rees-Garbutt et al., 2020b). Such approaches were recently used to further explore the minimal gene set of *M. florum* and compare it with JCVI-syn3.0 (Lachance et al., 2021). This required the reconstruction of a high-quality metabolic GEM for *M. florum*, consisting of 370 reactions, 208 genes, and 351 metabolites (*i*JL208). This model was experimentally validated using growth data on various sugars as well as gene expression and essentiality data, which were all in good agreement with the model predictions (Lachance et al., 2021). Gene essentiality data and metabolic constraints defined by the model allowed the prediction of a 562 kbp minimal genome containing 535 protein-coding genes. Since this prediction also considered the 387 previously identified *M. florum* transcription units (Matteau et al., 2020), its viability is more likely than previously mentioned hypothetical scenarios. Interestingly, this minimal genome contains 97 more protein-coding genes than JCVI-syn3.0, which could be due to real biological differences between the two organisms or simply be caused by prediction inaccuracies given the current gaps of knowledge in *M. florum* and Mollicutes biology. While this prediction shares 343 protein-coding genes with JCVI-syn3.0, it contains 129 genes unique to *M. florum* as well as 63 genes exclusively shared with JCVI-syn1.0, the parent strain of JCVI-syn3.0. This suggests that different minimal genome compositions probably exist, even for closely related species. However, most genes unique to *M. florum* are currently of unknown function, which complicates further investigation. Still, many protein-coding genes unique to *M. florum* or shared with JCVI-syn1.0 are associated with metabolic functions, notably transport and carbohydrate metabolism (Lachance et al., 2021). We can therefore imagine that different pathways could be used by minimal genomes to produce energy and fulfill cellular needs. Some minimal genome configurations could thus be more optimal than others. Indeed, 19 genes initially discarded in JCVI-syn3.0 were later reintroduced to resolve important morphological and growth defects, creating a more robust cell named JCVI-syn3A (Breuer et al., 2019; Pelletier et al., 2021). Among these genes, two are present in the minimal *M. florum* genome prediction. However, the construction of synthetic *M. florum* genomes will ultimately be needed to test and validate these computational predictions.

## Can we engineer the genome of *M. florum*?

The *M. florum* genome engineering toolbox is not as sophisticated as those available for *E. coli* or *Saccharomyces cerevisiae*. However, there is a growing number of methods that can be used for modifying the *M. florum* genome. Given its relative simplicity, Tn5 transposon mutagenesis was the first approach used in *M. florum* (Baby et al., 2018b). This system had previously been used in many bacterial species, including *M. mycoides* (Goryshin et al., 2000; Karas et al., 2014; Hutchison et al., 2016). Given the natural *M. florum* antibiotic susceptibility profile (Matteau et al., 2017), the widely used *tetM* gene conferring resistance to tetracycline was chosen as the selection marker in the transposon. The transformation of this transposon by electroporation resulted in tetracycline resistant *M. florum* colonies on ATCC 1161 plates (Baby et al., 2018b). Despite of a relatively high variability in the method efficiency, this allowed the creation of a collection comprising 2,806 individually picked transposon insertion mutants in which 430 of the 720 *M. florum* genes were found to be interrupted (Baby et al., 2018b; Lachance et al., 2021). Similar to the *E. coli* Keio collection (Baba et al., 2006), this library of gene-inactivated *M. florum* mutants represents an invaluable resource to study the biology of this near-minimal bacterium, especially for finding function to currently unassigned genes. This approach could also be repeated using different growth conditions to obtain additional information on the function of specific genes.

Another way to deliver genetic material into the genome is through the transformation of plasmids. Unfortunately, no natural plasmid has yet been reported to replicate in *M. florum*, and artificial plasmids developed in *M. mycoides*, *M. capricolum*, and *S. citri* have been shown to be incompatible with this species (Matteau et al., 2017). These plasmids harbor a partial or complete copy of the host chromosomal origin of replication (*oriC*) to replicate in their host. The *oriC* contains short DNA sequences known as DnaA boxes essential for the recognition by the DnaA protein, which is responsible for initiating DNA replication in bacteria (Messer, 2002). In Mollicutes, DnaA boxes are generally located within the two intergenic regions flanking the *dnaA* gene (Cordova et al., 2002; Lartigue et al., 2003; Ishag et al., 2017; Matteau et al., 2017). Artificial plasmids have recently been constructed using the *M. florum* predicted *oriC* (Matteau et al., 2017). The *tetM* gene was included in all tested *M. florum oriC* plasmids. While both intergenic regions surrounding the *dnaA* gene were shown to be essential for replication, contrasting with observations in *S. citri* (Lartigue et al., 2002), the presence of a copy of the *dnaA* gene was not. Plasmids containing both *dnaA* intergenic regions (pMflT-o3 and pMflT-o4) were stably maintained for more than 85 generations with or without antibiotics selection. Interestingly, *M. florum oriC* plasmids could successfully be transformed by electroporation or polyethylene glycol (PEG) transformation, as well as by conjugation from an *E. coli* strain using the RP4 system (Matteau et al., 2017). These plasmids allowed the validation of two additional selection markers, *pac* and *aadA1*, conferring resistance to puromycin and streptomycin/spectinomycin, respectively. While the *pac* marker had previously been used in other Mollicutes (Algire et al., 2009; Krishnakumar et al., 2010; Maglennon et al., 2013), this was the first reported use of the *aadA1* marker in a Mollicutes species. The functionality of this cassette also confirmed the recognition of P_*N25*_ promoter by the *M. florum* σ^70^ factor (Brunner and Bujard, 1987), which had not been used in the context of the *tetM* marker. This result is consistent with the sequence of the *M. florum* consensus promoter, which is, similar to *E. coli*, characterized by a strongly conserved−10 box of sequence TAWAAT (Matteau et al., 2020). However, in *M. florum*, the -35 box is highly degenerated. The *M. florum oriC* plasmids represent basic molecular tools that will help the validation of additional DNA parts in this bacterium, as well as facilitate the development of more sophisticated approaches to engineer its genome.

**FIGURE 2:**
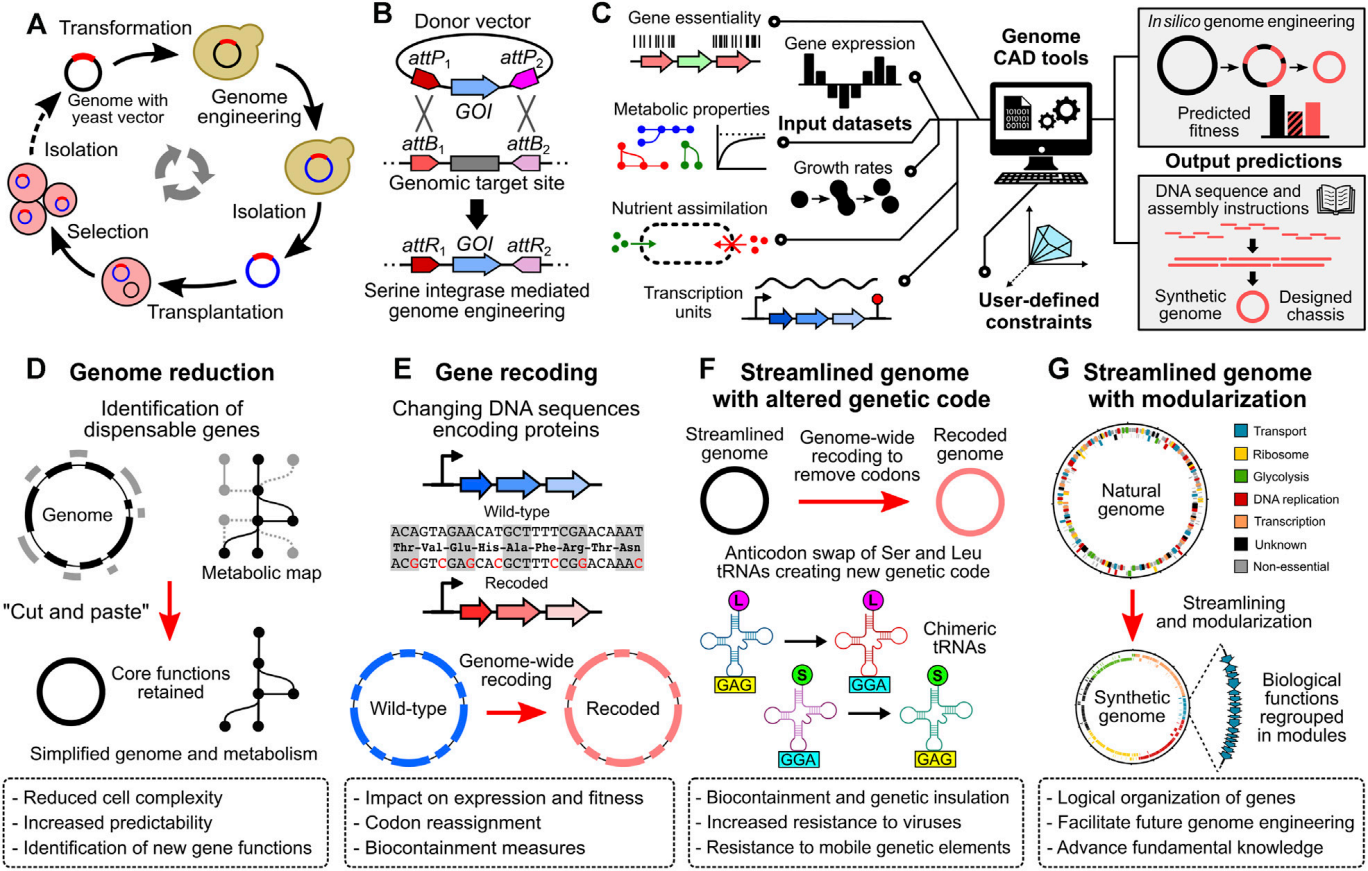
Genome engineering tools and projects to transform *Mesoplasma florum* into an optimized cell chassis for systems and synthetic biology. **(A)** Whole genome cloning and transplantation procedure. Bacterial genomes containing a yeast vector are first transformed in yeast to allow genetic modifications using the available molecular tools. Modified genomes are then carefully isolated and transplanted into a compatible recipient bacterium. Recipient cells adopt the phenotype conferred by the transplanted genome (see Lartigue et al., 2009; Labroussaa et al., 2019). **(B)** Serine integrase mediated genome engineering methodology. DNA fragments such as genes of interest (GOI) can be inserted or exchanged with the genome by the expression of a serine integrase. Serine integrases such as Bxb1 or PhiC31 catalyze the recombination between specific DNA sequences, namely, the *attP* and *attB* sites, resulting in *attL* and *attR* sites (*attL* sites not shown, see Merrick et al., 2018 for more details). **(C)** Integration of multiple data types by computer-aided design (CAD) tools to guide the design and optimize the synthesis, amplification, and assembly of large DNA fragments according to user-defined constraints. *In silico* models such as genome-scale models (GEMs) can be used to predict the fitness of designed genomes. **(D) to (G)** Example of synthetic genome projects. **(D)** Genome reduction, in which non-essential genes are removed from the genome, resulting in a reduced cell complexity and simplified metabolism. **(E)** Gene recoding at the genome scale. Within a given open reading frame, codons can be exchanged for synonymous ones, modifying the DNA sequence but not the corresponding amino acid sequence. **(F)** Streamlined genome using a swapped amino acid genetic code. By removing non-essential genes and recoding all remaining genes, specific codons can be removed from the genome, allowing anticodon swap between given tRNAs and creating an artificial genetic code. **(G)** Streamlined genome with gene of similar function regrouped in modules. Regrouping genes with related functions into modules can facilitate genome engineering efforts, and can be used to test specific hypotheses about gene regulation and genome organization.

Since *oriC* plasmids are replicated using the same mechanism as the chromosome, they are maintained at very low copy numbers in the cells. In *M. florum*, these plasmids are estimated to be present at 1 or 2 copies per cell (Matteau et al., 2017). In addition, their homology with the endogenous *oriC* region causes frequent recombination events with the host chromosome, a tendency also observed with *M. florum oriC* plasmids. While in some cases the integrated DNA cargo can interfere with the normal replication of the chromosome, this property can be exploited for genome engineering purposes. This was well demonstrated by the whole genome cloning (WGC) of the *M. florum* chromosome in the yeast *S. cerevisiae* (Labroussaa et al., 2016; Baby et al., 2018a) (Figure 2A). In that context, sequences enabling replication, partitioning, and selection in yeast were first introduced into the *M. florum* chromosome by the recombination of an *oriC* plasmid derivative. Following transformation in yeast, this allowed the *M. florum* chromosome to be replicated as a practically inert extrachromosomal element, with only minor impact on the yeast growth and cell cycle. WGC in yeast offers the opportunity to use the vast and well characterized molecular toolbox available in this model organism. For instance, the natural capacity of yeast to perform efficient homologous recombination was used to replace the duplicated *oriC* region resulting from the recombination of the *oriC* derivative plasmid by an *URA3* cassette (Baby et al., 2018a). Since many Mollicutes lack efficient molecular tools to modify their genome, WGC in yeast has been performed for several species, including *M. mycoides* and *M. genitalium* (Labroussaa et al., 2019). This procedure is at the heart of the strategy used to create JCVI-syn1.0 and JCVI-syn3.0 (Gibson et al., 2010; Hutchison et al., 2016). Whole genomes cloned and engineered in yeast must however be transplanted into a suitable recipient bacterium to assess their viability, a delicate procedure known as genome transplantation (Lartigue et al., 2007; Labroussaa et al., 2019) (Figure 2A). Due to its remarkable capacity to recognize the *oriC* region of other Mollicutes species, *M. capricolum* is generally used for this task (Lartigue et al., 2003; Lartigue et al., 2007; Labroussaa et al., 2016). Following transplantation and selection, the *M. capricolum* genome is replaced by the donor genome, and individual transplants can be recovered for validation and characterization. While many aspects of genome transplantation are still puzzling, the phylogenetic distance between the donor and recipient bacteria is known to play a critical role in the overall efficiency of the method (Labroussaa et al., 2016). Sharing ∼92% identity on the core proteome with *M. capricolum*, *M. florum* appears to be the most phylogenetically distant organism for which the transplantation with this recipient bacterium is possible (Baby et al., 2018a). Indeed, attempts to transplant the genome of *S. citri* and *S. floricola* have failed, and genome transplantation of more distant Mollicutes species such as *M. genitalium* and *M. hominis* has never been reported, albeit their genomes have been successfully cloned in yeast (Labroussaa et al., 2016; Labroussaa et al., 2019). Apart from the phylogenetic distance, other factors such as the concentration and quality of donor genomic DNA as well as the presence of mobile genetic elements or restriction-modification systems are also known to affect the success of the procedure (Lartigue et al., 2007; Gibson et al., 2010; Labroussaa et al., 2016; Labroussaa et al., 2019). The topology of the transplanted genomes might also be important as supercoiled DNA seems to drastically increase the transformability of large DNA molecules in *E. coli* (Mukai et al., 2020; Yoneji et al., 2021; Fujita et al., 2022).

## What could be the next steps in *M. florum* research?

Expanding the available molecular toolbox should certainly be one of the key priorities to fully harness the potential of *M. florum* for systems and synthetic biology. Even if the transplantation of the *M. florum* genome is possible (Figure 2A), the very low efficiency and high variability associated with this method using *M. florum* constitutes an important limitation to the in-yeast genome engineering strategy. It is not rare to obtain less than 10 *M. florum* transplants per experiment, or even no transplant at all (Labroussaa et al., 2016; Baby et al., 2018a). Further investigations are therefore required to enable rapid and easy prototyping of the *M. florum* genome cloned in yeast. Finding a new compatible recipient strain phylogenetically closer to *M. florum* than *M. capricolum* could in principle improve transplantation rates. Alternatively, targeted engineering of the recipient strain could also favor the recognition and boot-up of the transplanted genome. Nevertheless, genome transplantation remains a complex and delicate procedure. Complementary approaches should therefore be developed to facilitate the genetic modification of *M. florum*. Methods using serine integrases (Merrick et al., 2018) to efficiently exchange or insert DNA fragments at specific positions in the genome (Figure 2B) could prove very useful for *M. florum* since Tn*5* transposons insert randomly and current *oriC* plasmids tend to recombine only at the *oriC* region. Another option would be to adapt the well-known recombineering technique by properly expressing proteins of the λ-Red system (Datsenko and Wanner, 2000) or the GP35 recombinase, which was recently demonstrated to be functional in *M. pneumoniae* (Piñero-Lambea et al., 2020; Piñero-Lambea et al., 2022). This approach could even be coupled with the expression of the CRISPR-Cas9 system to further stimulate DNA recombination by cutting *M. florum*’s genome and counter-selecting unmodified or incorrectly repaired cells. Unlocking the CRISPR-Cas9 technology in *M. florum* would be a significant asset for future research on this bacterium, with a wide array of potential applications (Adli, 2018; Mariscal et al., 2018; Pickar-Oliver and Gersbach, 2019). Yet, heterologous proteins such as Cas9 must be sufficiently expressed in the host to display desired effect. On the other hand, constitutive or uncontrolled expression of many proteins is known to cause toxicity and can affect cell viability. Unfortunately, as of now not even a handful of promoters have been tested and validated on synthetic constructs introduced in *M. florum* (Matteau et al., 2017), none of which are inducible. Testing additional promoters -natural or synthetic- and combining them with other regulatory elements enabling strong activation or tight repression would unlock several methods (Kim et al., 2007; Breton et al., 2010; Domin et al., 2017; Etzel and Mörl, 2017; Ruegg et al., 2018; Piñero-Lambea et al., 2020). In addition, comparing these results with published transcriptional data would provide valuable information about the DNA sequences enabling strong transcription in this organism.

By increasing the molecular toolbox available in *M. florum*, performing large or extensive genome modifications and testing new hypotheses will become significantly easier. Combined with the most recent gene synthesis and high-throughput DNA assembly technologies (Gibson et al., 2010; Hughes and Ellington, 2017; Juhas and Ajioka, 2017; Schindler et al., 2018; Hoose et al., 2023), genome engineering projects could be undertaken (Figures 2D–G). For example, minimal genomes are powerful tools to study fundamental aspects of life, and constitute interesting cell chassis to learn genome design principles and develop promising applications in synthetic biology (Morowitz, 1984; Glass et al., 2017; Lachance et al., 2019). Their limited complexity increases predictability using modeling approaches and decreases the chance of unexpected interactions between artificial gene circuits and native host functions. Stripping the *M. florum* genome near its minimum would reduce the number of genes without any assigned function, and slightly decrease the costs associated with genome synthesis projects. Moreover, the comparison between a minimal *M. florum* genome and JCVI-syn3.0 could provide invaluable information about the different strategies used by bacteria to fulfill essential functions. Still, to enable rapid construction and testing of synthetic *M. florum* genomes, additional tools should be developed to integrate multiple data sources and properly guide the design as well as optimize the synthesis, amplification, and assembly of large DNA fragments (Figure 2C). With an efficient *M. florum* genome prototyping platform in hands, other exciting genome-wide projects could also become more realistic. Entire genome fractions could be recoded, separately or in combination with genome reduction efforts, to systematically investigate the impact of several parameters such as the GC content or the removal of internal transcription start sites (iTSSs) (Matteau et al., 2020) on gene expression and cell fitness (Figure 2E). Engineered or minimal *M. florum* cells will probably be sub-optimal at first, as observed with JCVI-syn3.0 and many other genome-reduced bacteria (Iwadate et al., 2011; Karcagi et al., 2016; Breuer et al., 2019; Pelletier et al., 2021; Dervyn et al., 2023). Artificial cells could next be subjected to adaptive laboratory evolution (ALE) for fine-tuning and selection of the most adapted mutants (Dragosits and Mattanovich, 2013; Sandberg et al., 2019). This strategy could be performed without adding any mutagenic compound or plasmid (Badran and Liu, 2015) given the particularly high DNA replication error rate of *M. florum* (Sung et al., 2012; Lynch et al., 2016). Interestingly, ALE experiments performed on JCVI-syn3A cultures led to growth rate improvements of >15%, corresponding to a doubling time of ∼80 min (Sandberg et al., 2023). The resulting *M. florum* mutants could be compared with ALE evolved JCVI-syn3A strains to see if they share similar mutation profiles and growth rates. Rare codons could also be systematically removed from the *M. florum* genome (Isaacs et al., 2011; Fredens et al., 2019), allowing codon reassignment and strict biocontainment measures. Artificial genetic codes could be developed and tested by swapping tRNA anticodons, thereby improving resistance to viruses and mobile genetic genetic elements (Zürcher et al., 2022; Nyerges et al., 2023) (Figure 2F). Genes with related functions could be regrouped into modules, reorganizing and streamlining the entire genome for engineering purposes (Hutchison et al., 2016; Coradini et al., 2020) (Figure 2G). Large genome portions could be inverted to study the importance of DNA orientation at large-scale. Every predicted transcriptional regulator could be tagged for genome-wide binding site assays, enabling high-throughput experimental determination of transcription regulation networks (Matteau and Rodrigue, 2015; Rossi et al., 2018). Protein sequences of entire pathways could be replaced by more or less phylogenetically related homologs to study protein compatibility and create chimeric genomes with enhanced properties. Guided by predictive tools such as the *i*JL208 GEM (Rees-Garbutt et al., 2020b; Rees-Garbutt et al., 2020a; Lachance et al., 2021), new metabolic capacities or biosynthetic pathways could be introduced by testing a large number of protein variants in parallel and finding the most optimal sequence combination for *M. florum* (Emanuel et al., 2017; Schubert et al., 2021). Synthetic genomics unlocks new possibilities that were simply not technically feasible not so long ago. As we move forward, the frontiers of biology will be redefined, allowing us to pursue and test hypotheses that long remained out of reach, thereby enhancing our comprehension of life at a deeper level.

## Abbreviations

GEM, Genome-scale model; PEG, Polyethylene glycol; *oriC*, Chromosomal origin of replication; WGC, Whole genome cloning; iTSS, Internal transcription start site; ALE, Adaptive laboratory evolution.
